# Isopropyl *ent*-15α-bromo-16-oxo­beyeran-19-oate

**DOI:** 10.1107/S1600536810002138

**Published:** 2010-01-23

**Authors:** Junqing Chen

**Affiliations:** a School of Chemistry and Chemical Engineering, Institute of Pharmaceutical Engineering, Southeast University, Nanjing 210096, People’s Republic of China

## Abstract

The title compound, C_23_H_35_BrO_3_, synthesized by esterification and bromination of isosteviol, comprises a fused four-ring system. Two of the six-membered rings adopt a regular chair conformation, whereas the remaining six-membered ring is an unsymmetrical distorted chair. The stereochemistry at the two six-membered ring junctions is *trans*, while the five-membered ring adopts an envelope conformation.

## Related literature

For the pharmacological activity of isosteviol, see: Liu *et al.* (2001 ▶); Mizushina *et al.* (2005 ▶); Wong *et al.* (2004 ▶); Xu *et al.* (2007 ▶). For ring conformations, see: Cremer & Pople (1975 ▶). For the synthesis of isosteviol derivates *via* esterification and bromination, see: Cai *et al.* (2009 ▶); Shi (2010 ▶); Wu *et al.* (2009 ▶).
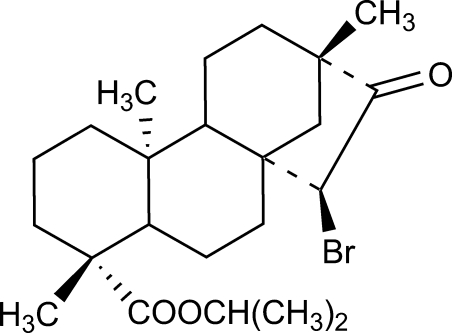


## Experimental

### 

#### Crystal data

C_23_H_35_BrO_3_
*M* *_r_* = 439.42
Orthorhombic, 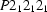

*a* = 11.203 (3) Å
*b* = 11.800 (3) Å
*c* = 16.988 (5) Å
*V* = 2245.8 (11) Å^3^
*Z* = 4
Mo *K*α radiation
μ = 1.85 mm^−1^
*T* = 298 K
0.50 × 0.45 × 0.41 mm

#### Data collection

Bruker SMART CCD area-detector diffractometer
Absorption correction: multi-scan (*SADABS*; Bruker, 1999 ▶) *T*_min_ = 0.458, *T*_max_ = 0.518
11796 measured reflections
3944 independent reflections
2383 reflections with *I* > 2σ(*I*)
*R*_int_ = 0.093

#### Refinement

*R*[*F* ^2^ > 2σ(*F* ^2^)] = 0.047
*wR*(*F* ^2^) = 0.108
*S* = 0.96
3944 reflections
245 parameters
H-atom parameters constrained
Δρ_max_ = 0.40 e Å^−3^
Δρ_min_ = −0.26 e Å^−3^
Absolute structure: Flack (1983 ▶), 1687 Friedel pairs
Flack parameter: −0.024 (13)

### 

Data collection: *SMART* (Bruker, 1999 ▶); cell refinement: *SAINT* (Bruker, 1999 ▶); data reduction: *SAINT*; program(s) used to solve structure: *SHELXS97* (Sheldrick, 2008 ▶); program(s) used to refine structure: *SHELXL97* (Sheldrick, 2008 ▶); molecular graphics: *SHELXTL* (Sheldrick, 2008 ▶); software used to prepare material for publication: *SHELXTL* and *PLATON* (Spek, 2009 ▶).

## Supplementary Material

Crystal structure: contains datablocks I, global. DOI: 10.1107/S1600536810002138/fj2269sup1.cif

Structure factors: contains datablocks I. DOI: 10.1107/S1600536810002138/fj2269Isup2.hkl

Additional supplementary materials:  crystallographic information; 3D view; checkCIF report

## Figures and Tables

**Table 1:** The puckering parameters (Å, °) for the six and five membered rings in compound I.

Puckering parameters	Ring *A*	Ring *B*	Ring *C*	Ring *D*
*Q*	0.540 (5)	0.563 (4)	0.641 (5)	0.441 (5)
*θ*	180.0 (5)	171.9 (4)	19.3 (4)	
*ϕ*	314 (67)	98 (3)	243.1 (14)	134.2 (7)

Ring *A* atoms C1–C5/C15, *B* C5–C6/C12–C15, *C* C6–C9/C12/C16 and *D* C9–C12/C16.

**Fig. 1.**
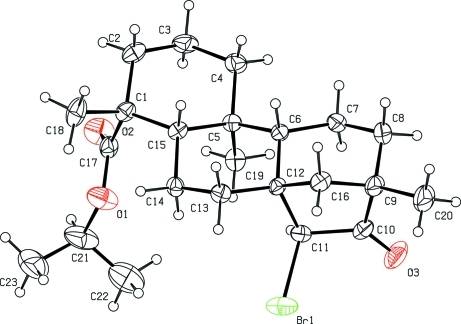
The molecular structure of the title compound with the atom-numbering scheme. Displacement ellipsoids are drawn at the 30% probability level.

## supplementary crystallographic information

### Comment

Isosteviol is a tetracyclic diterpenoid with a beyerane skeleton, which has good pharmacological activity against broad spectrum significant diseases including ischemia-reperfusion injury, hypertension, and cancer (Wong *et al.*, 2004; Liu, *et al.*, 2001; Xu, *et al.*, 2007; Mizushina *et al.*, 2005). The title compound was obtained by esterification and bromination of isosteviol, respectively. The molecule structure of (I) contains a fused four-ring system *A*/*B*/*C*/*D* (Fig. 1). The *A*/*B* ring and *B*/*C* junction are *trans*-fused, and *C*/*D* is *cis*-fused. Rings *A* and *B* adopt chair conformations and ring *C* is in a distorted chair conformation, with puckering amplitude *Q* = 0.641 (5) Å, θ= 19.3 (4)° and φ= 243.1 (14)° (Cremer & Pople, 1975). The distortion may be attributed to the narrowing of the C9—C16—C12 bond angle to 103.8 (4)°. The five-membered ring *D* adopts an envelope conformation with atom C16 displaced from the C9/C10/C11/C12 plane by 0.281 (5) Å. The C17—C1—C2—C3 torsion angle of -73.8 (5)° describes the *β*-orientation of the isopropyl ester group with respect to the *ent*-kaurane nucleus.

### Experimental

Isosteviol was obtained by hydrolysis of stevioside with 10% sulfuric acid at 95 °C for 7 h and recrystallization from ethanol gave colorless crystals of isosteviol in 80% yield. Isosteviol (5 g, 16 mmol), K_2_CO_3_ (3.3 g, 32 mmol) and 2-bromopropane (2 ml, 21 mmol) were dissolved in 100 ml acetonitrile. After reflux of the above mixture for 5.5 h, the resulting mixture was cooled to room temperature, and then distilled to one third volume under reduced pressure. The residue was poured into ice water, and the resulting precipitates were collected by filtration. The crude product was purified by recrystallization with ethanol to afford isopropyl *ent*-16-oxobeyeran-19-oate (5.4 g, 95%).

Isopropyl *ent*-16-oxobeyeran-19-oate (3.6 g, 10 mmol) and *N*-bromosuccinimide (5.3 g, 30 mmol) were dissolved in 50 ml tetrachloromethane. After reflux of the above mixture for 11.5 h, the resulting mixture was cooled to room temperature, and then distilled under reduced pressure. The residue was purified by column chromatography on silica (petroleum ether/ethyl acetate = 60:1, *v*/*v*) to give the title compound (4.0 g, 91%). Crystals of the title compound suitable for X-ray diffraction were obtained by slow evaporation of ethanol solution at room temperature. m.p. 396–397 K; ^1^H NMR(500 MHz, CDCl_3_), δ_H_ p.p.m.: 0.79(s, 3H), 1.10(s, 3H), 1.19(s, 3H), 1.24–1.26(q, 6H), 1.09–2.21(m, 18H), 4.51–4.52(d, 1H, *J*=2.63 Hz), 4.96–5.01(m, 1H); ^13^C NMR(75 MHz, CDCl_3_), δ_C_ p.p.m.: 13.84, 18.91, 19.99, 20.70, 20.85, 21.63, 21.70, 28.86, 37.84, 37.89, 38.48, 38.85, 39.50, 43.01, 43.72, 48.35, 50.06, 56.01, 56.10, 57.20, 67.38, 176.46, 215.73; MS (ESI): [*M*+H]^+^ 439.2, [*M*+K]^+^ 477.1; IR(KBr): ν 2954.56, 1743.42, 1716.42 cm^-1^; Anal. calcd for C_23_H_35_BrO_3_ (%): C 62.87, H 8.03, Found: C 62.81, H 7.89.

### Refinement

All H atoms were placed in geometrical positions and constrained to ride on their parent atoms with C–H distances in the range 0.96–0.98 Å, and included in the final cycles of refinement using a riding model, with *U*_iso_(H) = 1.5*U*_eq_(C) for methyl H and 1.2*U*_eq_(C) for other H atoms.

### Crystal data

**Table:**

C_23_H_35_BrO_3_	*D*_x_ = 1.300 Mg m^−^^3^
*M**_r_* = 439.42	Melting point = 396–397 K
Orthorhombic, *P*2_1_2_1_2_1_	Mo *K*α radiation, λ = 0.71073 Å
Hall symbol: P 2ac 2ab	Cell parameters from 2087 reflections
*a* = 11.203 (3) Å	θ = 2.2–17.6°
*b* = 11.800 (3) Å	µ = 1.85 mm^−^^1^
*c* = 16.988 (5) Å	*T* = 298 K
*V* = 2245.8 (11) Å^3^	Block, colourless
*Z* = 4	0.50 × 0.45 × 0.41 mm
*F*(000) = 928	

### Data collection

**Table:**

Bruker SMART CCD area-detector diffractometer	3944 independent reflections
Radiation source: fine-focus sealed tube	2383 reflections with *I* > 2σ(*I*)
graphite	*R*_int_ = 0.093
phi and ω scans	θ_max_ = 25.0°, θ_min_ = 2.1°
Absorption correction: multi-scan (*SADABS*; Bruker, 1999)	*h* = −13→12
*T*_min_ = 0.458, *T*_max_ = 0.518	*k* = −9→14
11796 measured reflections	*l* = −20→19

### Refinement

**Table:**

Refinement on *F*^2^	Hydrogen site location: inferred from neighbouring sites
Least-squares matrix: full	H-atom parameters constrained
*R*[*F*^2^ > 2σ(*F*^2^)] = 0.047	*w* = 1/[σ^2^(*F*_o_^2^) + (0.0375*P*)^2^] where *P* = (*F*_o_^2^ + 2*F*_c_^2^)/3
*wR*(*F*^2^) = 0.108	(Δ/σ)_max_ < 0.001
*S* = 0.96	Δρ_max_ = 0.40 e Å^−^^3^
3944 reflections	Δρ_min_ = −0.26 e Å^−^^3^
245 parameters	Extinction correction: *SHELXL97* (Sheldrick, 2008), Fc^*^=kFc[1+0.001xFc^2^λ^3^/sin(2θ)]^-1/4^
0 restraints	Extinction coefficient: 0.0190 (12)
Primary atom site location: structure-invariant direct methods	Absolute structure: Flack (1983), 1687 Friedel pairs
Secondary atom site location: difference Fourier map	Flack parameter: −0.024 (13)

### Special details

**Table:**

Geometry. All e.s.d.'s (except the e.s.d. in the dihedral angle between two l.s. planes) are estimated using the full covariance matrix. The cell e.s.d.'s are taken into account individually in the estimation of e.s.d.'s in distances, angles and torsion angles; correlations between e.s.d.'s in cell parameters are only used when they are defined by crystal symmetry. An approximate (isotropic) treatment of cell e.s.d.'s is used for estimating e.s.d.'s involving l.s. planes.
Refinement. Refinement of *F*^2^ against ALL reflections. The weighted *R*-factor *wR* and goodness of fit *S* are based on *F*^2^, conventional *R*-factors *R* are based on *F*, with *F* set to zero for negative *F*^2^. The threshold expression of *F*^2^ > σ(*F*^2^) is used only for calculating *R*-factors(gt) *etc*. and is not relevant to the choice of reflections for refinement. *R*-factors based on *F*^2^ are statistically about twice as large as those based on *F*, and *R*- factors based on ALL data will be even larger.

### Fractional atomic coordinates and isotropic or equivalent isotropic displacement parameters (Å^2^)

**Table:**

	*x*	*y*	*z*	*U*_iso_*/*U*_eq_	
Br1	0.68536 (6)	0.37853 (5)	0.23962 (3)	0.0740 (3)	
O1	0.8434 (3)	0.8337 (3)	0.2930 (2)	0.0733 (12)	
O2	0.9186 (4)	0.9167 (3)	0.3975 (2)	0.0694 (12)	
O3	0.7310 (4)	0.2287 (3)	0.3988 (3)	0.0794 (13)	
C1	0.7080 (4)	0.8666 (4)	0.3967 (2)	0.0385 (12)	
C2	0.7046 (5)	0.9117 (4)	0.4811 (3)	0.0498 (14)	
H2A	0.7466	0.9836	0.4828	0.060*	
H2B	0.6221	0.9260	0.4954	0.060*	
C3	0.7588 (5)	0.8336 (4)	0.5414 (3)	0.0541 (15)	
H3A	0.7474	0.8655	0.5935	0.065*	
H3B	0.8439	0.8273	0.5320	0.065*	
C4	0.7018 (5)	0.7149 (4)	0.5382 (2)	0.0459 (13)	
H4A	0.7434	0.6656	0.5748	0.055*	
H4B	0.6194	0.7203	0.5553	0.055*	
C5	0.7051 (4)	0.6609 (3)	0.4559 (2)	0.0323 (11)	
C6	0.6208 (4)	0.5550 (3)	0.4581 (3)	0.0320 (11)	
H6	0.5446	0.5848	0.4774	0.038*	
C7	0.6560 (5)	0.4652 (4)	0.5190 (3)	0.0475 (14)	
H7A	0.7369	0.4404	0.5083	0.057*	
H7B	0.6552	0.4995	0.5709	0.057*	
C8	0.5740 (5)	0.3617 (4)	0.5195 (3)	0.0526 (14)	
H8A	0.5003	0.3811	0.5464	0.063*	
H8B	0.6121	0.3012	0.5489	0.063*	
C9	0.5444 (5)	0.3191 (4)	0.4363 (3)	0.0486 (14)	
C10	0.6638 (5)	0.3074 (4)	0.3954 (3)	0.0487 (14)	
C11	0.6880 (5)	0.4191 (4)	0.3513 (2)	0.0381 (12)	
H11	0.7664	0.4493	0.3659	0.046*	
C12	0.5894 (4)	0.5006 (3)	0.3769 (2)	0.0307 (11)	
C13	0.5508 (4)	0.5921 (4)	0.3191 (3)	0.0375 (12)	
H13A	0.5434	0.5588	0.2671	0.045*	
H13B	0.4728	0.6201	0.3344	0.045*	
C14	0.6371 (4)	0.6909 (4)	0.3149 (2)	0.0354 (12)	
H14A	0.6073	0.7466	0.2778	0.042*	
H14B	0.7139	0.6643	0.2961	0.042*	
C15	0.6524 (4)	0.7460 (3)	0.3957 (2)	0.0318 (11)	
H15	0.5704	0.7580	0.4140	0.038*	
C16	0.4857 (4)	0.4155 (4)	0.3910 (3)	0.0433 (14)	
H16A	0.4223	0.4500	0.4216	0.052*	
H16B	0.4530	0.3888	0.3415	0.052*	
C17	0.8350 (5)	0.8739 (4)	0.3649 (3)	0.0427 (12)	
C18	0.6337 (5)	0.9479 (4)	0.3449 (3)	0.0606 (17)	
H18A	0.5518	0.9463	0.3616	0.091*	
H18B	0.6386	0.9243	0.2909	0.091*	
H18C	0.6643	1.0235	0.3499	0.091*	
C19	0.8353 (4)	0.6305 (4)	0.4370 (3)	0.0418 (12)	
H19A	0.8396	0.5977	0.3854	0.063*	
H19B	0.8642	0.5770	0.4751	0.063*	
H19C	0.8834	0.6977	0.4388	0.063*	
C20	0.4736 (5)	0.2087 (4)	0.4388 (4)	0.0731 (19)	
H20A	0.3994	0.2211	0.4657	0.110*	
H20B	0.5189	0.1520	0.4662	0.110*	
H20C	0.4580	0.1835	0.3861	0.110*	
C21	0.9562 (6)	0.8432 (6)	0.2497 (4)	0.088 (2)	
H21	1.0143	0.8872	0.2801	0.105*	
C22	1.0009 (6)	0.7247 (7)	0.2372 (5)	0.110 (3)	
H22A	1.0197	0.6911	0.2872	0.166*	
H22B	1.0712	0.7265	0.2050	0.166*	
H22C	0.9403	0.6806	0.2116	0.166*	
C23	0.9293 (9)	0.9009 (7)	0.1744 (4)	0.131 (3)	
H23A	0.9020	0.9766	0.1848	0.197*	
H23B	0.8682	0.8598	0.1469	0.197*	
H23C	1.0001	0.9039	0.1426	0.197*	

### Atomic displacement parameters (Å^2^)

**Table:**

	*U*^11^	*U*^22^	*U*^33^	*U*^12^	*U*^13^	*U*^23^
Br1	0.0767 (4)	0.0953 (5)	0.0501 (4)	0.0172 (4)	0.0068 (3)	−0.0263 (3)
O1	0.068 (3)	0.097 (3)	0.055 (2)	−0.043 (2)	0.011 (2)	−0.0233 (19)
O2	0.047 (3)	0.091 (3)	0.070 (3)	−0.024 (2)	−0.014 (2)	−0.016 (2)
O3	0.080 (3)	0.040 (2)	0.118 (4)	0.021 (2)	0.023 (3)	0.001 (2)
C1	0.038 (3)	0.035 (3)	0.043 (3)	−0.001 (2)	−0.009 (2)	−0.006 (2)
C2	0.049 (4)	0.041 (3)	0.059 (3)	−0.005 (2)	−0.006 (3)	−0.017 (2)
C3	0.062 (4)	0.062 (4)	0.038 (3)	−0.010 (3)	−0.004 (3)	−0.020 (3)
C4	0.052 (4)	0.050 (3)	0.036 (3)	−0.004 (3)	−0.005 (3)	−0.004 (2)
C5	0.028 (3)	0.038 (3)	0.031 (2)	0.006 (2)	−0.005 (2)	−0.0012 (19)
C6	0.028 (3)	0.033 (3)	0.035 (3)	0.009 (2)	0.004 (2)	0.002 (2)
C7	0.063 (4)	0.044 (3)	0.035 (3)	0.011 (3)	0.005 (3)	0.003 (2)
C8	0.067 (4)	0.040 (3)	0.051 (3)	0.001 (3)	0.013 (3)	0.010 (3)
C9	0.053 (4)	0.033 (3)	0.060 (4)	−0.012 (3)	0.017 (3)	−0.004 (3)
C10	0.057 (4)	0.033 (3)	0.056 (3)	0.008 (3)	0.007 (3)	−0.007 (3)
C11	0.037 (3)	0.043 (3)	0.034 (3)	−0.002 (2)	0.004 (3)	−0.006 (2)
C12	0.028 (3)	0.027 (3)	0.038 (3)	−0.001 (2)	0.004 (2)	−0.002 (2)
C13	0.036 (3)	0.041 (3)	0.036 (3)	−0.004 (2)	−0.006 (2)	−0.004 (2)
C14	0.037 (3)	0.036 (3)	0.033 (3)	0.000 (2)	−0.007 (2)	0.004 (2)
C15	0.024 (3)	0.033 (3)	0.037 (3)	−0.002 (2)	−0.008 (2)	−0.007 (2)
C16	0.033 (3)	0.038 (3)	0.058 (3)	−0.006 (2)	0.011 (2)	−0.007 (3)
C17	0.053 (4)	0.033 (3)	0.042 (3)	−0.011 (3)	−0.010 (3)	0.000 (2)
C18	0.063 (4)	0.040 (3)	0.079 (4)	0.004 (3)	−0.030 (3)	0.007 (3)
C19	0.027 (3)	0.046 (3)	0.051 (3)	−0.001 (3)	−0.007 (2)	0.006 (2)
C20	0.081 (5)	0.043 (4)	0.095 (5)	−0.014 (3)	0.025 (4)	0.004 (3)
C21	0.072 (4)	0.121 (6)	0.070 (5)	−0.053 (4)	0.033 (4)	−0.027 (5)
C22	0.078 (5)	0.155 (8)	0.097 (6)	−0.012 (5)	0.032 (4)	0.005 (6)
C23	0.186 (10)	0.113 (7)	0.094 (6)	−0.046 (6)	0.064 (6)	0.004 (5)

### Geometric parameters (Å, °)

**Table:**

Br1—C11	1.957 (4)	C10—C11	1.539 (6)
O1—C17	1.314 (5)	C11—C12	1.528 (6)
O1—C21	1.467 (6)	C11—H11	0.9800
O2—C17	1.199 (5)	C12—C13	1.522 (6)
O3—C10	1.197 (6)	C12—C16	1.554 (6)
C1—C17	1.524 (6)	C13—C14	1.515 (6)
C1—C2	1.529 (6)	C13—H13A	0.9700
C1—C18	1.546 (6)	C13—H13B	0.9700
C1—C15	1.554 (6)	C14—C15	1.528 (6)
C2—C3	1.506 (7)	C14—H14A	0.9700
C2—H2A	0.9700	C14—H14B	0.9700
C2—H2B	0.9700	C15—H15	0.9800
C3—C4	1.541 (6)	C16—H16A	0.9700
C3—H3A	0.9700	C16—H16B	0.9700
C3—H3B	0.9700	C18—H18A	0.9600
C4—C5	1.536 (6)	C18—H18B	0.9600
C4—H4A	0.9700	C18—H18C	0.9600
C4—H4B	0.9700	C19—H19A	0.9600
C5—C19	1.536 (6)	C19—H19B	0.9600
C5—C15	1.551 (6)	C19—H19C	0.9600
C5—C6	1.567 (6)	C20—H20A	0.9600
C6—C7	1.532 (6)	C20—H20B	0.9600
C6—C12	1.562 (6)	C20—H20C	0.9600
C6—H6	0.9800	C21—C23	1.481 (10)
C7—C8	1.528 (6)	C21—C22	1.500 (9)
C7—H7A	0.9700	C21—H21	0.9800
C7—H7B	0.9700	C22—H22A	0.9600
C8—C9	1.537 (7)	C22—H22B	0.9600
C8—H8A	0.9700	C22—H22C	0.9600
C8—H8B	0.9700	C23—H23A	0.9600
C9—C10	1.514 (7)	C23—H23B	0.9600
C9—C16	1.523 (7)	C23—H23C	0.9600
C9—C20	1.526 (7)		
			
C17—O1—C21	120.0 (4)	C13—C12—C6	110.0 (3)
C17—C1—C2	109.6 (4)	C11—C12—C6	110.3 (4)
C17—C1—C18	105.4 (4)	C16—C12—C6	107.3 (4)
C2—C1—C18	107.7 (4)	C14—C13—C12	113.3 (4)
C17—C1—C15	115.0 (4)	C14—C13—H13A	108.9
C2—C1—C15	108.6 (4)	C12—C13—H13A	108.9
C18—C1—C15	110.2 (4)	C14—C13—H13B	108.9
C3—C2—C1	114.5 (4)	C12—C13—H13B	108.9
C3—C2—H2A	108.6	H13A—C13—H13B	107.7
C1—C2—H2A	108.6	C13—C14—C15	110.9 (4)
C3—C2—H2B	108.6	C13—C14—H14A	109.4
C1—C2—H2B	108.6	C15—C14—H14A	109.4
H2A—C2—H2B	107.6	C13—C14—H14B	109.4
C2—C3—C4	111.4 (4)	C15—C14—H14B	109.4
C2—C3—H3A	109.3	H14A—C14—H14B	108.0
C4—C3—H3A	109.3	C14—C15—C5	111.1 (3)
C2—C3—H3B	109.3	C14—C15—C1	116.4 (4)
C4—C3—H3B	109.3	C5—C15—C1	115.7 (3)
H3A—C3—H3B	108.0	C14—C15—H15	103.9
C5—C4—C3	113.6 (4)	C5—C15—H15	103.9
C5—C4—H4A	108.8	C1—C15—H15	103.9
C3—C4—H4A	108.8	C9—C16—C12	103.8 (4)
C5—C4—H4B	108.8	C9—C16—H16A	111.0
C3—C4—H4B	108.8	C12—C16—H16A	111.0
H4A—C4—H4B	107.7	C9—C16—H16B	111.0
C4—C5—C19	108.0 (4)	C12—C16—H16B	111.0
C4—C5—C15	108.8 (3)	H16A—C16—H16B	109.0
C19—C5—C15	112.0 (4)	O2—C17—O1	121.7 (5)
C4—C5—C6	107.2 (4)	O2—C17—C1	126.1 (4)
C19—C5—C6	113.0 (3)	O1—C17—C1	112.1 (4)
C15—C5—C6	107.6 (3)	C1—C18—H18A	109.5
C7—C6—C12	111.7 (3)	C1—C18—H18B	109.5
C7—C6—C5	114.4 (4)	H18A—C18—H18B	109.5
C12—C6—C5	116.3 (3)	C1—C18—H18C	109.5
C7—C6—H6	104.3	H18A—C18—H18C	109.5
C12—C6—H6	104.3	H18B—C18—H18C	109.5
C5—C6—H6	104.3	C5—C19—H19A	109.5
C8—C7—C6	113.7 (4)	C5—C19—H19B	109.5
C8—C7—H7A	108.8	H19A—C19—H19B	109.5
C6—C7—H7A	108.8	C5—C19—H19C	109.5
C8—C7—H7B	108.8	H19A—C19—H19C	109.5
C6—C7—H7B	108.8	H19B—C19—H19C	109.5
H7A—C7—H7B	107.7	C9—C20—H20A	109.5
C7—C8—C9	112.7 (4)	C9—C20—H20B	109.5
C7—C8—H8A	109.1	H20A—C20—H20B	109.5
C9—C8—H8A	109.1	C9—C20—H20C	109.5
C7—C8—H8B	109.1	H20A—C20—H20C	109.5
C9—C8—H8B	109.1	H20B—C20—H20C	109.5
H8A—C8—H8B	107.8	O1—C21—C23	107.0 (6)
C10—C9—C16	102.5 (4)	O1—C21—C22	106.7 (5)
C10—C9—C20	113.2 (4)	C23—C21—C22	112.0 (6)
C16—C9—C20	115.3 (5)	O1—C21—H21	110.3
C10—C9—C8	105.2 (4)	C23—C21—H21	110.3
C16—C9—C8	108.3 (4)	C22—C21—H21	110.3
C20—C9—C8	111.5 (5)	C21—C22—H22A	109.5
O3—C10—C9	127.2 (5)	C21—C22—H22B	109.5
O3—C10—C11	125.2 (5)	H22A—C22—H22B	109.5
C9—C10—C11	107.6 (4)	C21—C22—H22C	109.5
C12—C11—C10	105.9 (4)	H22A—C22—H22C	109.5
C12—C11—Br1	114.7 (3)	H22B—C22—H22C	109.5
C10—C11—Br1	105.1 (3)	C21—C23—H23A	109.5
C12—C11—H11	110.3	C21—C23—H23B	109.5
C10—C11—H11	110.3	H23A—C23—H23B	109.5
Br1—C11—H11	110.3	C21—C23—H23C	109.5
C13—C12—C11	117.9 (4)	H23A—C23—H23C	109.5
C13—C12—C16	110.2 (4)	H23B—C23—H23C	109.5
C11—C12—C16	100.2 (3)		

### Table 1 The puckering parameters (Å, ° ) for the six and five membered rings in compound I.

**Table:**

Puckering parameters	Ring *A*	Ring *B*	Ring *C*	Ring *D*
*Q*	0.540 (5)	0.563 (4)	0.641 (5)	0.441 (5)
*θ*	180.0 (5)	171.9 (4)	19.3 (4)	
*φ*	314 (67)	98 (3)	243.1 (14)	134.2 (7)

Ring *A* atoms C1–C5/C15, *B* C5–C6/C12–C15, *C* C6–C9/C12/C16 and *D* C9–C12/C16.
